# A New Arthritis Therapy with Oxidative Burst Inducers

**DOI:** 10.1371/journal.pmed.0030348

**Published:** 2006-09-12

**Authors:** Malin Hultqvist, Peter Olofsson, Kyra A Gelderman, Jens Holmberg, Rikard Holmdahl

**Affiliations:** Medical Inflammation Research, Lund University, Sweden; Universiteit Amsterdam, Netherlands

## Abstract

**Background:**

Despite recent successes with biological agents as therapy for autoimmune inflammatory diseases such as rheumatoid arthritis (RA), many patients fail to respond adequately to these treatments, making a continued search for new therapies extremely important. Recently, the prevailing hypothesis that reactive oxygen species (ROS) promote inflammation was challenged when polymorphisms in *Ncf1,* that decrease oxidative burst, were shown to increase disease severity in mouse and rat arthritis models. Based on these findings we developed a new therapy for arthritis using oxidative burst-inducing substances.

**Methods and Findings:**

Treatment of rats with phytol (3,7,11,15-tetramethyl-2-hexadecene-1-ol) increased oxidative burst in vivo and thereby corrected the effect of the genetic polymorphism in arthritis-prone *Ncf1* ^DA^ rats. Importantly, phytol treatment also decreased the autoimmune response and ameliorated both the acute and chronic phases of arthritis. When compared to standard therapies for RA, anti-tumour necrosis factor-α and methotrexate, phytol showed equally good or better therapeutic properties. Finally, phytol mediated its effect within hours of administration and involved modulation of T cell activation, as injection prevented adoptive transfer of disease with arthritogenic T cells.

**Conclusions:**

Treatment of arthritis with ROS-promoting substances such as phytol targets a newly discovered pathway leading to autoimmune inflammatory disease and introduces a novel class of therapeutics for treatment of RA and possibly other chronic inflammatory diseases.

## Abstract 2

Treatment of arthritis in rats with phytol, a reactive oxygen species promoting substance, suggests a novel pathway of autoimmune inflammatory disease and possibly a novel therapeutic strategy.

## Editors' Summary

**Background.:**

Rheumatoid arthritis (RA) is a chronic illness that affects between 0.3% and 1% of people worldwide, causing pain and swelling in joints, tendons, and other tissues, and frequently leading to permanent deformity and disability. RA involves an abnormal attack by cells of the immune system against the body's own connective tissues (so-called autoimmunity). Current drugs for RA work by counteracting the molecules that cause the pain and swelling (inflammation). By reducing the severity of autoimmune inflammation, these drugs may also reduce the disease's long-term damage to joints.

Inflammation is not always abnormal, but in fact plays an important part in the body's defense against infection. As part of their activity against disease-causing bacteria, the white blood cells known as granulocytes generate reactive oxygen species (ROS), sometimes known as “free radicals.” After engulfing invading bacteria, neutrophils release an “oxidative burst” of ROS—essentially the subcellular equivalent of pouring hydrogen peroxide on a wound to disinfect it. A complex of molecules known collectively as the NADPH oxidase complex has the specific function of generating ROS to fuel the oxidative burst. Interestingly, recent experiments in arthritis-prone rats found that animals with an altered form of one of the subunits of this complex, Ncf1, that decreased the production of ROS also had greater susceptibility to arthritis. This finding was surprising because free radicals have generally been associated with inflammation and long-term damage to cells, so that a reduction in ROS might have been expected to decrease susceptibility to an inflammatory disease like RA.

**Why Was This Study Done?:**

Because many patients with autoimmune inflammatory illnesses like RA do not respond to currently available therapies, new approaches to treatment merit investigation. Based on the observed association between reduced ROS and increased susceptibility to arthritis, the researchers wanted to find out whether treatment with a compound that increases ROS production by the NADPH oxidase complex would cause an improvement in arthritis.

**What Did the Researchers Do and Find?:**

The researchers tested a compound called phytol in arthritis-prone rats to see how it affected inflammation. It is known that arthritis can be induced in these rats by injecting them with an oil called pristane. The researchers found that phytol caused a strong oxidative burst in human granulocyte cells grown in the laboratory, but did not cause arthritis in rats; whereas pristane, which does cause arthritis, caused a lower oxidative burst in the granulocytes.

They then studied whether phytol prevented arthritis in rats. They found that rats injected with phytol were protected from arthritis following a later injection of pristane. Given this result, they wanted to know if phytol increased ROS in the rats as it did in laboratory cell cultures. Studying granulocytes taken from rats that had been treated with phytol, they found that the oxidative burst of these cells was indeed increased, and remained increased for several weeks after treatment. They went on to test phytol as a treatment for active arthritis, and found that it dramatically reduced swollen joints and destruction of cartilage when given to rats with acute pristane-induced arthritis.

The beneficial effects of phytol were seen not only in rats bred with a form of Ncf1 that produces abnormally low amounts of ROS, but also in rats whose granulocytes produce normal oxidative bursts. When compared (in rats) to drugs licensed for RA (etanercept and methotrexate), phytol appeared to be at least as effective. The activity of phytol against arthritis was shown to involve T lymphocytes, as injection of phytol inhibited transfer of pristane-induced arthritis with these cells.

**What Do These Findings Mean?:**

These experiments raise the intriguing possibility of an entirely new modality for treating autoimmune diseases; namely, through drugs designed to increase the production of ROS. This study raises a number of practical and scientific issues. For example, it is not known whether reduced capacity to produce ROS is a significant factor in human RA. Also, the connection between ROS production (by granulocytes) and autoimmune arthritis (which involves activity by T lymphocytes) remains to be clarified. Finally, the destructive effects typically associated with free radicals (such as damage to DNA and blockage of blood vessels) could complicate the use of this approach in humans, and like any new drugs, those that increase ROS production might have other, unanticipated side effects. Whatever the outcome of drug development efforts, however, this study is an excellent reminder that there are no “good” or “evil” biochemicals—in the intricacies of cellular metabolism, it's all a matter of balance.

**Additional Information.:**

**Please access these Web sites via the online version of this summary at** http://dx.doi.org/10.1371/journal.pmed.0030348.

- The Arthritis Foundation: Rheumatoid Arthritis pages
- Medical Inflammation Research pages (R. Holmdahl research group)
- Wikipedia chapter on Rheumatoid Arthritis (note: Wikipedia is a free Internet encyclopedia that anyone can edit)
- Wikipedia chapter on Reactive Oxygen Species (note: Wikipedia is a free Internet encyclopedia that anyone can edit)

## Introduction

Rheumatoid arthritis (RA) is a complex chronic inflammatory disease dependent on multiple interacting environmental and genetic factors, making it difficult to understand its pathogenesis and thereby to find effective therapies. The therapy of RA is mainly based on methotrexate (MTX) and biological agents that directly target molecules involved in the pathogenesis of RA—e.g., the human soluble tumour necrosis factor receptor etanercept (Enbrel). However, cytokines targeted with such biological agents also play a general role in the immune defence, and the risk of infections increases with use of these agents [1]. Therefore, a continued search for successful therapies for RA is extremely important.

Disease-causing mechanisms underlying RA that can be targeted with a preventive or therapeutic treatment have been difficult to identify [2]. In addition, the search for genes predisposing for autoimmunity has so far had limited success [3–6]. Recently, *neutrophil cytosolic factor 1 (Ncf1)* was identified by positional cloning as a gene controlling arthritis severity in rats [7]. Ncf1 (alias p47phox) is an important subunit of the NADPH oxidase complex responsible for the one-electron reduction of oxygen that ultimately yields reactive oxygen species (ROS) [8,9]. This oxidative burst process is used by phagocytes primarily to kill invading pathogens [10]. The surprising finding with the *Ncf1* polymorphism in the susceptible Dark Agouti (DA) rat was that increased susceptibility to arthritis was associated with low oxidative burst capacity [7], contradicting the hypothesis in current use that high levels of free radicals promotes inflammation [11]. This finding led us to propose that substances that increase the activation of the NADPH oxidase complex could ameliorate and possibly prevent severe arthritis. In this study we used rats as an experimental model of RA to identify compounds that increase oxidative burst capacity in vivo and investigate whether these substances thereby could have a therapeutic effect on arthritis.

## Methods

### Animals

Rats of strains DA and LEW.1F (Zentralinstitut für Versuchstierzucht, Hannover, Germany) were kept in a climate-controlled environment with 12 h light/dark cycles, housed in polystyrene cages containing wood shavings, and fed standard rodent chow and water ad libitum in the animal house of Medical Inflammation Research (http://www.inflam.lu.se). The rats were found to be free from common pathogens including Sendai virus, Hantaan virus, coronavirus, reovirus, cytomegalovirus, and Mycoplasma pulmonalis. The DA.*Ncf1^E3^* and DA.*pia34* strains have been described [7,12]. The experiments were approved by local (Malmö/Lund, Sweden) ethical committee license M70/01 and M70/04.

### Human Promyelocytes

The human promyelocyte line HL-60 (CCl-240; ATCC, Manassas, Virginia, United States) was cultured in D-MEM (Gibco, Paisley, UK) complemented with Hepes, 5% fetal calf serum, and penicillin-streptomycin at standard cell culture concentrations. The cells were differentiated to granulocytes by culture in the presence of 1.25% DMSO (Sigma-Aldrich, St. Louis, Missouri, United States) for 6 d [13]. Before they were assayed the cells were washed and resuspended in D-PBS (Gibco) to a concentration of 10^7^ cells/ml.

### Oxidative Burst Assay of Granulocytes In Vitro

Saturated alkane molecules (C8-C17) (Larodan Fine Chemicals AB, Malmö, Sweden), pristane (2,6,10,14-tetramethylpentadecane), and phytol (3,7,11,15-Tetramethyl-2-hexadecen-1-ol) (all from Sigma-Aldrich) were tested for oxidative burst-inducing capacity according to a previously described method [14]. Oils were solubilized by dilution at 1%–5% concentration in 10% β-cyclodextrin (Sigma-Aldrich) in PBS. β-cyclodextrin by itself had no stimulating effect on ROS production. Briefly, 5 μl of resuspended oils were added to 96-well plates containing 5 × 10^5^ cells/well in a total volume of 200 μl of PBS containing isoluminol and horseradish peroxidase (final isoluminol concentration 100 mg/ml; Sigma-Aldrich) and horseradish peroxidase type II (5 units/ml; Sigma-Aldrich). Samples were gently mixed and data collection was initiated immediately. Extracellular ROS production was followed at 37 °C as luminescence signal (FluoStar Optima, BMG Labtechnologies, Offenburg, Germany) and presented as maximal relative signal during a measurement period of 30 min.

### Induction and Evaluation of Arthritis

Disease was induced in all rats at the age of 6–12 wk. Rats were sex- and age-matched within all experiments.

Pristane-induced arthritis (PIA) and oil-induced arthritis was induced by a subcutaneous (SC) injection at the base of the tail with 200 μl of pristane or alkane (C8–C17) oils. Collagen-induced arthritis (CIA) was induced by a SC injection of 100 μl of rat collagen type II (CII) (100–150 μg/rat), purified from the Swarm rat chondrosarcoma as previously described [15], emulsified in IFA (Difco, BD Diagnostic Systems, Maryland, United States).

Nonoil collagen-induced arthritis (NOCIA) was induced by emulsifying rat CII (300 μg) in a mixture of LPS (50 μg; Sigma-Aldrich), CPG (5′-TCC ATG ACG TTC CTG ACG TT-3′) (45 μg; MWG-Biotech AG, Ebersberg, Germany), and alum (6 mg; Sigma-Aldrich) and injected SC in a total volume of 300 μl.

Arthritis development was monitored with a macroscopic scoring system of the four limbs ranging from 0 to 15 (one point for each swollen or red toe, one point for midfoot digit or knuckle, and five points for a swollen ankle). The scores of the four paws were added, yielding a maximum total score of 60 for each rat [16].

### Treatment of Arthritis

Unless stated otherwise, preventive treatment of arthritis was performed by SC injections of 200 μl of phytol, C11, or C16 5 d before induction of arthritis. Control rats were left untreated.

Therapeutic treatments were administered SC or intraperitoneally (IP; 200 μl) after onset of disease in the acute or chronic phase.

Comparisons to standard treatments were performed by injection of phytol (200 μl SC), etanercept (generously provided by Prof. H. Burkhart, Erlangen, Germany; 0.4 mg/kg in PBS SC), or MTX (Wyeth Lederle, Solna, Sweden; 0.1–0.25 mg MTX/kg rat IP) on days 8, 10, and 12 in a total volume of 200 μl.

Treatment of adoptive transfer was performed by injecting 200 μl of phytol SC in the recipients 3 h before or 3 or 5 and 8 d after transfer. Donor rats were treated with 500 μl of phytol SC on days −5, 0, 3, 7, or 10 (3 h before spleen isolation).

### Determination of Oxidative Burst Activity Ex Vivo

The level of intracellular oxidative burst ex vivo was measured by preparing single-cell suspensions from blood, spleen, draining lymph nodes (LNs), or bone marrow (BM). Red blood cells were lysed with ammonium chloride (pH 7.4) at a concentration of 0.84%.

Oxidative burst in granulocytes and T cells was determined by incubation of cells for 30 min at 4 °C with biotin-labelled antibody HIS-48 (anti-granulocytes) or PerCP-labelled R73 antibody (anti-T cell receptor) (BD Biosciences Pharmingen, San Jose, California, United States). After they were washed with PBS, cells were incubated with allophycocyanin-conjugated streptavidin (BD Pharmingen) for 20 min at 4 °C. To determine the level of NADPH activity we used a modified version of the oxidative burst activity flow cytometry assay previously described [17]. Briefly, cells were resuspended in Dulbecco's complete medium without FCS after staining, and incubated for 10 min at 37 °C with 3 μM dihydrorhodamine-123 (Molecular Probes, Leiden, The Netherlands), which, after oxidization by hydrogen peroxide (H_2_O_2_), peroxinitrite (ONOO^−^) and hydroxyl radicals (OH•) to rhodamine-123, emits a bright fluorescent signal upon excitation by blue light. Cells were then stimulated with 200 ng/ml phorbol 12-myristate 13-acetate (PMA; Sigma-Aldrich) for 20 min at 37 °C. After a wash with PBS they were acquired on a FACSort (BD Biosciences), gated on cell-type. R-123 fluorescence intensity was measured on FL-1 and results expressed in relative fluorescence units.

### Lipid Peroxidation

Blood was collected in nonheparinized tubes and serum was isolated by centrifugation. The level of lipid peroxidation was measured by a modified version of the malonedialdehyde-thiobarbituric acid method previously described [18]. In brief, a 1:1:1 mixture containing thiobarbituric acid (3.75g/l; Sigma-Aldrich), TCA (15%), and HCl (0.33 M) was freshly prepared. 3 μl of 2,6-di-tert-butyl-4-methylphenol (2% in ethanol) was added to 100 μl of serum together with 200 μl of the reaction mix and mixed vigorously. The samples were then incubated for 25 min at 95 °C and centrifuged at 13,000 rpm for 5 min. The absorbance of the supernatant was then measured at 536 nm in a Spectra Max (Molecular Devices, Sunnyvale, California, United States).

### Ex Vivo Analysis of Lymphocyte Populations

Blood, spleen, and draining LNs were taken from rats 5 d after SC injection with 200 μl of phytol, and single-cell suspensions were prepared. Red blood cells were lysed in 0.84% ammonium chloride (pH 7.4) and remaining cells were washed in PBS before staining with antibodies OX-33 (anti-CD45RA), OX-35 (anti-CD4), 341 (anti-CD8b), and R73 (anti-T cell receptor) (all from BD Pharmingen). Cells were acquired on a FACSort (BD Biosciences) and B-to-T cell ratio as well as CD4/CD8 ratio were determined after gating on respective cell type.

### Total IgG and IgM Levels

In order to determine total antibody level, plates (MaxiSorp, Nunc, Roskilde, Denmark) were coated with anti-IgG (Zymed, San Francisco, California, United States) or anti-IgM (BD Pharmingen) in PBS overnight. After plates were blocked for 2 h at room temperature with 1% BSA (Sigma-Aldrich), plasma diluted in PBS was added. Levels of antibodies were then detected using biotinylated anti-IgG (Zymed) or anti-IgM antibody (BD Pharmingen) followed by incubation with peroxidase-conjugated streptavidin and ABTS tablets (Roche Diagnostics GmbH, Mannheim, Germany). Results were analyzed in a Spectra Max at OD 405 nm (Molecular Devices). The relative amount of antibodies was compared to a pooled positive control.

### Ex Vivo Cell Death Assay

Blood, spleens, and draining LNs were taken from DA.*Ncf1* ^DA^ rats 3 h or 5 d after they were injected intradermally with 200 μl of phytol. Single-cell suspensions were prepared and red blood cells lysed with 0.84% ammonium chloride (pH 7.4). Cells were washed with PBS, then stained with OX-35 (anti-CD4) (BD Pharmingen) for 30 min at 4 °C. Cells were resuspended in 200 μl of PBS containing 5 μl of Annexin V (BD Pharmingen) and 1 μg/ml of propidium iodide (PI) (Molecular Probes) after washing. Cells were acquired on a FACSort (BD Biosciences) and the percentages of apoptotic (Annexin V^+^ and PI^−^) and necrotic (Annexin V^+^ and PI^+^) CD4^+^ cells were determined.

### Antibody Response against CII

Antibodies titres against CII in plasma were determined by ELISA in 96-well plates (MaxiSorp), coated overnight at 4 °C with 10 μg/ml rat CII in 50 μl of PBS per well. All washing was performed using PBS containing 0.1% Tween 20. The plasma was diluted in PBS and analyzed in duplicates. The amounts of bound IgG antibodies were estimated after incubation with biotin-conjugated isotype-specific antibodies (Zymed) followed by peroxidase-conjugated streptavidin and ABTS (Roche Diagnostics) as substrate followed by detection in a Spectra Max at OD405 nm (Molecular Devices). The relative amount of antibodies in plasma was determined in comparison with a positive control of pooled serum.

### Delayed-Type Hypersensitivity in response to CII

On day 67 after NOCIA immunization, rats were challenged with 20 μg of CII in 0.05 M acetic acid injected into the epidermis of the right external ear. Acetic acid was injected in the left ear as a control. The delayed-type hypersensitivity (DTH) response was measured after 24 h as the difference in swelling between the right and left ear in percent.

### Determination of Serum Levels of Cartilage Oligomeric Matrix Protein

The plasma concentration of cartilage oligomeric matrix protein (COMP) was determined by a competitive ELISA as described previously [19]. Briefly, rat COMP was used to coat the microtitre plates and to prepare the standard curve included in each plate. Plates were blocked with 1% BSA in PBS before adding plasma. COMP levels were then detected by using a rabbit polyclonal antiserum against rat COMP (generously provided by Professor Dick Heinegård, Lund, Sweden). The amounts of serum COMP were estimated after incubation with an alkaline phosphatase-conjugated, isotype-specific antibody (DAKO, Glostrup, Denmark) and a phosphatase substrate (Sigma-Aldrich), followed by detection in a SpectraMax (Molecular Devices) at OD 405 nm.

### Reversion of the Phytol Effect with Histamine Dihydrochloride

Rats were injected with 200 μl of phytol SC on day 25 after the induction of disease with an injection of 200 μl of pristane. At day 35 half of the phytol-treated rats were injected with histamine dihydrochloride (Sigma-Aldrich) both SC (125 μl of 0.6 mg/ml) and IP (125 μl of 0.6 mg/ml) for a total of 0.15 mg of histamine dihydrochloride/rat. The same rats were injected again in the same way on day 40 after pristane. The control rats were left untreated.

### Histological Analysis

DA.*Ncf1* ^DA^ rats were injected either with phytol and pristane or with pristane alone on day 0 (200 μl SC). Half of the pristane-injected rats were treated with 200 μl of phytol SC day 13. All rats were sacrificed day 22 and paws were taken for histological analysis. The samples were fixed in 4% phosphate-buffered paraformaldehyde before being decalcified with 1% EDTA and embedded in paraffin. Sections were stained with erythrosine-hematoxylin and magnified 20×.

### Adoptive Transfer of Arthritogenic Spleen Cells

DA.*Ncf1* ^DA^ rat spleens were collected and homogenized 10–14 d after injection of 500 μl of pristane. The red blood cells were lysed in 0.84% ammonium chloride (pH 7.4) and remaining cells were washed with PBS before culture in D-MEM (Gibco) containing concanavalin A (Sigma-Aldrich) for 44 h. After they were washed and resuspended in PBS the cells were injected IP into naïve or phytol-treated DA rats (35 × 10^6^ cells/rat).

### Statistics

Quantitative data are expressed as mean ± standard error of the mean, and significance analysis was performed using Mann-Whitney test. All results were compared to those from the control group unless otherwise indicated.

## Results

### Oxidative Burst-Inducing Agents Prevent Arthritis

**Figure 1:**
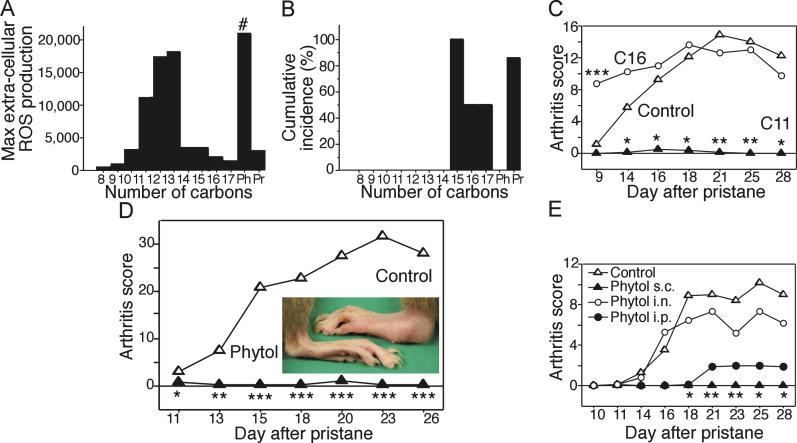
Substances Inducing Oxidative Burst In Vitro Prevent Arthritis (A) Structure of the oil is important for oxidative burst-inducing effect in vitro. Extracellular ROS production was measured with an isoluminol assay on HL-60 cells after in vitro stimulation with alkanes, phytol (Ph), or pristane (Pr). Data represent one of several experiments. ^#^Value is divided by 10. (B) Alkanes longer than C15 induce arthritis. Arthritogenicity of alkanes (*n* = 4), phytol (Ph; *n* = 5), and pristane (Pr; *n* = 7) after SC administration in DA.*Ncf1* ^DA^ rats. Results are presented as cumulative incidence in percent. (C) Oxidative burst-inducing alkanes prevent arthritis. Arthritis-preventing effect of selected alkanes after SC administration day −5 before PIA induction (control, *n* = 17; C11, *n* = 6; and C16, *n* = 5) in DA.*Ncf1* ^DA^ rats. (D) Phytol inhibits development of arthritis. Preventive effect of phytol on PIA in DA.*Ncf1* ^DA^ rats. Phytol was injected SC day −5 prior to pristane injection (control, *n* = 12; phytol, *n* = 13). (E) The preventive effect of phytol is dependent on route of administration. Preventive effect of phytol on PIA in DA.*Ncf1* ^DA^ rats after IP (200 μl), SC (200 μl), or intranasal (25 μl) administration on days −5 and +5 (*n* = 7). *p < 0.05, **p < 0.01, ***p < 0.001.

Since low oxidative burst capacity increased arthritis susceptibility [7], we proposed that substances elevating ROS production could have therapeutic effects on arthritis. We had already seen that certain oils with an alkane structure, such as phytol (3,7,11,15-tetramethyl-2-hexadecen-1-ol) and pristane (2,6,10,14-tetramethylpentadecane), had an oxidative burst-inducing capacity in vitro*.* Additionally, despite structural similarity, pristane induced arthritis, whereas phytol protected against it [7]. To examine the structure/function relationship we screened a number of short saturated alkanes (C8–C17) using an isoluminol-based oxidative burst assay [14] on human promyelocytes (HL-60) differentiated to granulocytes in the presence of DMSO. Alkanes 11–13 carbons long were the most potent in activating the NADPH oxidase complex (Figure 1A). To investigate their arthritogenic potential, we injected the alkanes into arthritis-susceptible DA rats carrying the DA *Ncf1* allele (*Ncf1* ^DA^). Alkanes with 15 carbons or more, such as pristane, induced arthritis, whereas shorter alkanes did not (Figure 1B). We therefore concluded that the arthritis-inducing effect of these oils is independent of their oxidative burst-inducing capacity.

Next we wanted to see if oxidative burst-inducing effect correlated with arthritis prevention using PIA [20] as a model of human RA. C11, shown to induce oxidative burst in vitro, was potent in reducing arthritis severity when administered 5 d before arthritis induction (Figure 1C). Similar results were also seen with C12 and C13 (unpublished data). In contrast, C16, which was arthritogenic and did not increase oxidative burst, did not protect against arthritis (Figure 1C). These results clearly demonstrate that small changes in structure determine the effect of the molecule. Since phytol was superior in inducing oxidative burst in vitro as well as in preventing arthritis when injected before induction of disease (Figure 1A and 1D), it was chosen for further studies of efficacy.

We then investigated the dependency of arthritis-preventing effects on route of administration. When phytol was administered SC, IP, or intranasally 5 d before and after PIA induction it was evident that SC administration resulted in the best preventive effect (Figure 1E).

### Phytol Increases Oxidative Burst Capacity In Vivo

**Figure 2:**
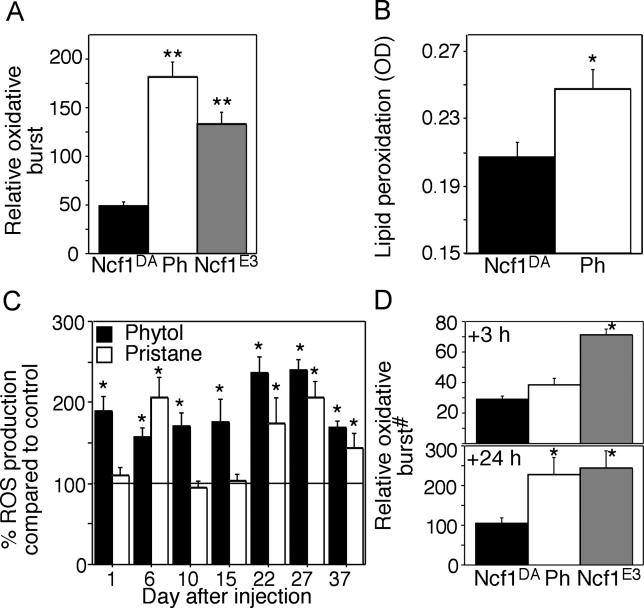
Phytol Increases Oxidative Bursts In Vivo (A) Phytol (Ph) restores the oxidative burst in vivo. Oxidative burst response to PMA stimulation in vitro in spleen granulocytes taken 5 d after SC phytol injection in DA.*Ncf1* ^DA^ rats (200 μl) or from naïve DA.*Ncf1* ^E3^ compared to naïve DA.*Ncf1* ^DA^ (*n* = 5). (B) Increased levels of products from lipid peroxidation in serum after phytol injection. Sera were taken 5 d after SC phytol injection in DA.*Ncf1* ^DA^ and analyzed for levels of malondialdehyde, which reflects lipid peroxidation (*n* = 4). (C) Phytol injection has a long-term effect on oxidative burst capacity of granulocytes. Capacity of DA.*Ncf1* ^DA^ blood granulocytes to exert an oxidative burst in response to PMA stimulation in vitro after SC injection of phytol or pristane (200 μl) on day 0. Values are presented as percent of oxidative burst compared naïve DA.*Ncf1* ^DA^ rats (represented by the line) (*n* = 5). (D) Phytol increases the oxidative burst capacity in BM granulocytes. Oxidative burst capacity of granulocytes from BM taken 3 hours (*n* = 5) and 24 hours (*n* = 4) after SC phytol (Ph; 200 μl) injection in DA.*Ncf1* ^DA^ rats or from naïve DA.*Ncf1* ^E3^, compared to naive DA.*Ncf1* ^DA^. ^#^Experiments were performed independent of each other and levels should thus not be compared. *p < 0.05, **p < 0.01.

We also wanted to know whether phytol has the same oxidative burst-inducing effect in vivo*.* In particular, we asked whether phytol would correct the low oxidative burst capacity seen in rats carrying the *Ncf1* ^DA^ allele. We analyzed spleen granulocytes 5 d after phytol injection for intracellular oxidative burst response to PMA stimulation in vitro. Phytol clearly increased the oxidative burst capacity in DA.*Ncf1* ^DA^ rats to the level of the DA.*Ncf1* ^E3^ congenic rats carrying the arthritis-protective E3 *Ncf1* allele (Figure 2A). Serum levels of malondialdehyde, the main product of lipid peroxidation, were also increased after phytol injection (Figure 2B), reflecting a higher level of oxidation in vivo.

Since a single phytol injection mediated a surprisingly long suppression of arthritis (Figure 1D) we investigated whether this protective effect correlated with a sustained increase in oxidative burst capacity. Thus, we followed the oxidative burst response to PMA in blood granulocytes for 7 wk after injection of either pristane or phytol. In order to avoid intraexperiment variations, results were normalized to a naïve standard group. An increase in oxidative burst capacity was evident the day after phytol (but not after pristane injection) and persisted for several weeks (Figure 2C). During development of arthritis the PMA response of pristane-injected rats increased compared to naïve rats, possibly reflecting a systemic response to the local inflammation; this finding agrees with reports of oxygen radicals in synovial neutrophils in human RA [21].

One possible explanation for the long-lasting effect of phytol could be that the phenotype of cell populations with longer lifespans than those of neutrophilic granulocytes, such as stem cells in BM, is altered. To address this hypothesis we analyzed BM cells from DA.*Ncf1* ^DA^ rats 3 and 24 h after phytol. No effect of injection was seen after 3 h, but 24 h after administration of phytol the oxidative burst capacity was elevated up to the level of the protected *DA.Ncf1^E3^* rats (Figure 2D).

### Increased Oxidative Burst Alters Cell Distribution

**Figure 3:**
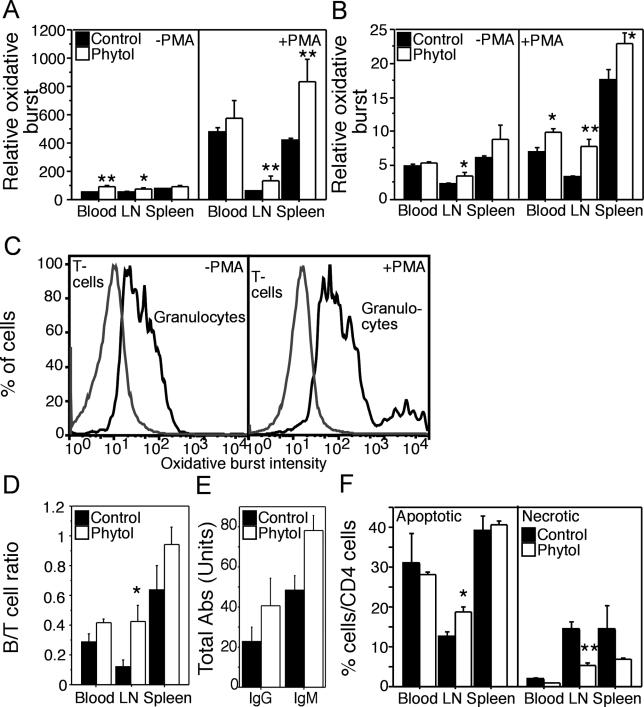
Increased Levels of ROS Alter Cell Distributions (A) The phytol-induced increase in oxidative burst in granulocytes is not organ specific. Granulocytes taken from blood, LNs, and spleen on day 5 after phytol injection were analyzed for oxidative burst with and without PMA stimulation (*n* = 5). (B) Phytol increases oxidative burst in T cells with or without PMA stimulation 5 d after injection (*n* = 5). (A) and (B) are taken from different experiments and levels should thus not be compared. (C) ROS production in T cells is low compared to that in granulocytes. ROS production with and without PMA stimulation in spleen granulocytes and T cells from a naïve DA.*Ncf1* ^DA^ rat. (D) Cell distribution is altered after phytol. Cell distribution in different organs analyzed 5 d after phytol by staining for B and T cells (*n* = 5). (E) The phytol effect is not mediated by a general immune-suppressive effect. Total levels of IgG and IgM antibodies in plasma measured 5 d after phytol injection expressed as relative values (*n* = 5). (F) Phytol does not increase cell death. Percentage of apoptotic (Annexin V+ and PI−) and necrotic (Annexin V+ and PI+) CD4^+^ cells ex vivo 5 d after phytol injection (*n* = 5). *p < 0.05, **p < 0.01.

Next we investigated the effect of phytol in vivo in blood, spleen, and draining LNs. Five days after injection, cells from the different anatomical sites were collected and analyzed for oxidative burst. Phytol clearly increased the background activation of granulocytes. Also after PMA stimulation, phytol-primed granulocytes had a higher response capacity (Figure 3A), whereas T cells showed only a barely detectable increase in ROS production (Figure 3B). Compared to the level of oxidative burst in granulocytes, T cells are minor producers of ROS (Figure 3C). T cells were also stimulated with plate-bound anti-CD3, but this did not further increase the ROS production (Figure S1). The increase in T cell oxidative burst could be a reflection of contaminating ROS from granulocytes. B/T cell ratio was altered after phytol injection (Figure 3D), presumably through an increase of B cell number, because a tendency towards higher levels of IgG and IgM was also seen (Figure 3E). The same tendency was seen after phytol treatment in PIA (unpublished data). Alternatively, the increased B/T ratio could have resulted from decreased T cell numbers. Phytol did not significantly alter T cell distribution, as no effect on the ratio of CD4/CD8 was seen (unpublished data). To investigate whether the change in cell distribution was due to phytol toxicity we analyzed the percentage of apoptotic and necrotic T cells ex vivo 5 d after injection. Levels of dead T cells did not increase (Figure 3F), suggesting that the increased B/T ratio or the disease-preventing effect is not mediated via increased depletion of T cells.

### Phytol Suppresses CIA and CII Autoimmunity

**Figure 4:**
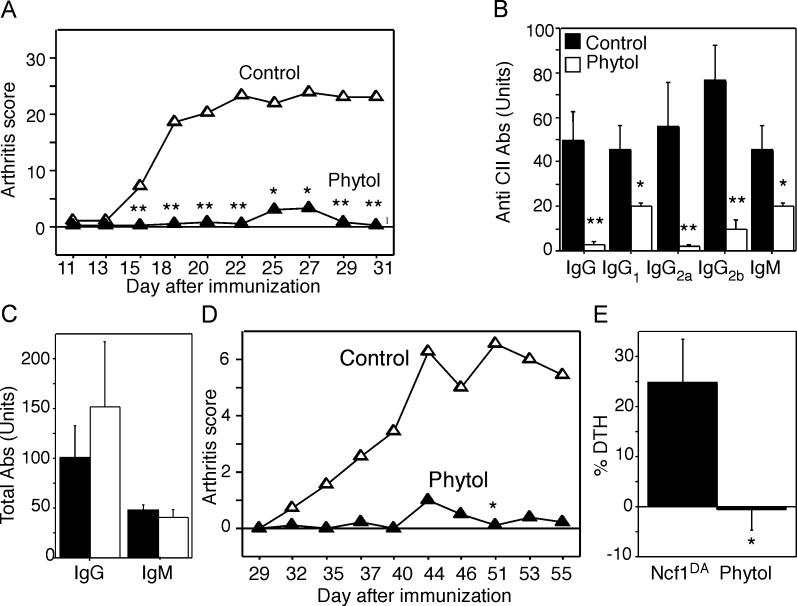
Phytol Suppresses Type II Collagen Autoimmunity (A) Phytol prevents collagen-induced arthritis in DA.*Ncf1* ^DA^ rats. Effect of SC administration of phytol (200 μl) 5 d before immunization with CII (*n* = 7). (B) Phytol decreases anti-CII antibody levels in plasma. At day 27 after immunization, plasma samples were taken and analyzed for anti-CII IgG and IgM levels (*n* = 7). Titres are relative to pooled positive plasma (units). (C) Phytol does not decrease total antibody levels. Total levels of IgG and IgM in plasma taken day 27 after immunization compared to pooled positive plasma (*n* = 7). (D) Phytol reduces arthritis severity also in NOCIA, an arthritis model induced without involvement of oil. Preventive effect on arthritis of phytol injection (200 μl SC) 5 d prior to induction of NOCIA in DA.*Ncf1* ^DA^ rats (control *n* = 7; phytol *n* = 8). (E) DTH response is abolished after phytol injection. Day 67 after NOCIA immunization the immune system was challenged by an injection of CII in the ear. Subsequent increase in ear volume was measured and compared to control ear (control *n* = 7; phytol *n* = 8). *p < 0.05, **p < 0.01.

Since PIA is a model with a unique pathogenesis involving αβT cells, operating in the effector phase as well [20,22], we analyzed whether phytol operates also in CIA [23], a model in which antibodies are important in the joint inflammatory attack [24]. A significant decrease in arthritis severity comparable to the effect on PIA was observed after preventive administration of phytol also in CIA (Figure 4A). Importantly, phytol suppressed the autoimmune response, as plasma levels of CII specific antibodies on day 27 after immunization were reduced (Figure 4B). Levels of anti-CII of all isotypes were decreased after phytol treatment; also IgM response was lower (Figure 4B), suggesting no shift in T cell response. However, total IgG and IgM levels were not affected (Figure 4C), arguing for a specific effect of phytol on CII-reactive cells. As IgG anti-CII response is T cell-dependent, these data indicate a lower activity of CII autoreactive T helper cells.

### Phytol Inhibits Arthritis Induced without Adjuvant Oil

So far, arthritis was induced with an oil component such as pristane or IFA in the CII immunization. To exclude a role of oil in the induction of disease we developed a new model, NOCIA. CII was emulsified in a mixture of nonoil adjuvants, such as alum, lipopolysaccharide, and a bacterial DNA sequence (CpG) and injected SC. Phytol injection before immunization completely blocked the development of disease (Figure 4D). In addition, when the immune system was challenged at the end of experiment (day 67 after NOCIA induction) by a CII injection in the ear, we found that phytol totally inhibited the DTH response (Figure 4E).

### Phytol Decreases Arthritis Severity in Established Disease

**Figure 5:**
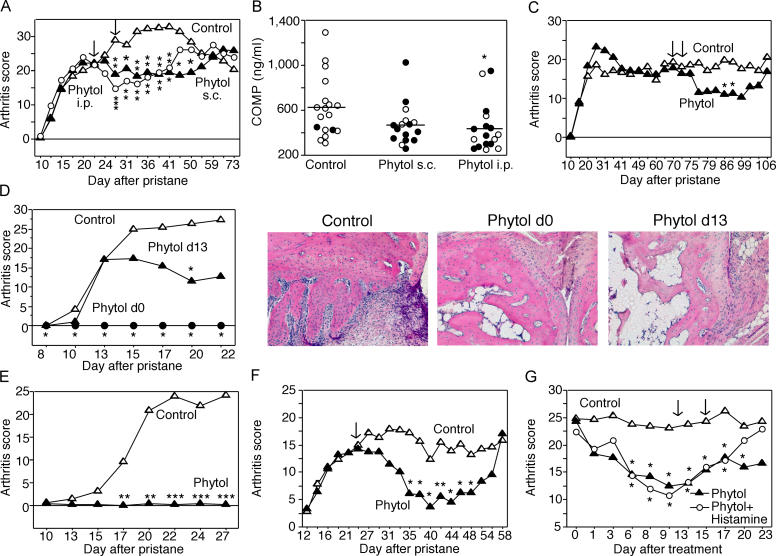
Phytol Suppresses Established Arthritis (A) Phytol injection ameliorates acute arthritis. Therapeutic effect of phytol in acute phase of PIA in DA.*Ncf1* ^DA^ rats after two injections (200 μl) 5 d apart (arrows) starting at the estimated peak of disease (day 22). (Significance indications above line represent SC administration and below IP administration) (control *n* = 18, IP *n* = 17, and SC *n* = 16). (B) Cartilage destruction is decreased after phytol injection. Plasma were taken day 38 and analyzed for COMP levels as a measurement of cartilage destruction. Results are presented as circles, where each circle represents one individual and filled circles represent rats with a mean reduction in score after day of first treatment (day 22). Lines represent mean values. (C) Phytol decreases arthritis severity in the chronic phase of arthritis. Phytol was injected IP twice (200 μl) (arrows), starting day 70 after pristane injection in DA.*Ncf1* ^DA^ rats with 3 d between treatments (control, *n* = 19; phytol, *n* = 18). (D) Phytol affects macroscopic and microscopic inflammation. Pristane-injected rats treated with phytol on either day 0 or day 13 (*n* = 4). On day 22 rats were sacrificed and paws taken for histology and stained with erythrosine-hematoxylin (photomicrographs to right of graph; 20× magnification). (E) The preventive effect of phytol is not dependent on genetic background. Arthritis-preventing effect of phytol when injected (200 μl SC) 5 d before induction of PIA in LEW.1F rats (*n* = 9). (F) Phytol has an arthritis-ameliorating effect in rats that have a functional oxidative burst. Rats heterozygous for the functional *Ncf1* (*Ncf1* ^E3^) allele with an arthritis susceptibility locus on Chromosome 6 were injected IP (200 μl) with phytol at the estimated peak of disease (day 24, arrow; control, *n* = 13; phytol, *n* = 15). (G) Histamine dihydrochloride reverses the ameliorating effect of phytol, possibly by blocking oxidative burst. Injection of histamine twice (200 μl SC and IP), with 5 d between injections (arrows), starting 10 d after phytol treatment (day 26 after pristane) in DA.*Ncf1* ^DA^ rats could reverse the arthritis-ameliorating effect (*n* = 5). Results are presented as difference in score compared to day of treatment (day 0). *p < 0.05, **p < 0.01, ***p < 0.001.

To be considered as a new candidate therapy for RA in humans, phytol needs to be therapeutically effective in established arthritis. Thus we tested the efficacy of phytol in both acute and chronic phases of PIA. To test phytol in the acute phase, rats were treated twice with 5 d between injections, starting at the peak of the disease (around day 22). Phytol injection markedly decreased arthritis severity (Figure 5A). A decrease in circulating COMP—a measurement of ongoing cartilage destruction [19]—occurred after treatment (Figure 5B). To determine the therapeutic efficiency in the chronic phase of PIA, phytol was injected two times with 3 d between injections starting when the disease has passed the first phase and relapses were observed (around day 70). The chronic phase is quite different from the acute phase and is controlled by a different set of genes [25]. Importantly, a significant decrease in arthritis severity in treated rats was observed (Figure 5C). Our results clearly show that phytol not only has preventive effects but is therapeutic all along the disease course. Histopathologic analysis of paws from rats treated either before or during the acute phase of PIA revealed an effect on both inflammation and joint destruction (Figure 5D). In sections taken from rats treated in the acute phase of disease a mild inflammation could be seen together with remnants of earlier, more severe inflammation. Rats treated before onset showed no signs of inflammation.

### Phytol-Mediated Arthritis Suppression Is Not Restricted to the DA Background or the *Ncf1* ^DA^ Allele

To investigate whether the phytol effect was dependent on the unique DA genetic background, we induced arthritis in LEW.1F rats, which also develop a chronic arthritis with high incidence [26]. LEW.1F rats have a lower oxidative burst than do the protected DA.*Ncf1^E3^* rat, caused by the same *Ncf1* allele that is carried by DA.*Ncf1* ^DA^ [7]. As with DA.*Ncf1* ^DA^ rats, LEW.1F rats were also protected from arthritis after a preventive phytol injection (Figure 5E).

Treatment with oxidative burst inducers was thus far done by increasing ROS production in DA.*Ncf1* ^DA^ rats. Next we wanted to investigate whether rats expressing the *Ncf1* ^E3^ allele could be treated successfully with phytol. Since it has been shown that one allele of *Ncf1* ^E3^ is enough to restore the oxidative burst and to prevent arthritis in DA.*Ncf1* ^DA^ rats [7], we used a DA double congenic rat strain expressing not only *Ncf1* ^E3/DA^ but also a congenic fragment on Chromosome 6 allowing arthritis development [12]. Also in these rats, phytol treatment in acute phase of disease resulted in a significant decrease in disease severity (Figure 5F). These are important findings indicating that phytol can also be therapeutic in individuals with a “normal” oxidative burst capacity.

### Phytol-Induced Suppression of Arthritis Is Reversible

An important issue for a new therapy is whether the effect is reversible. We selected histamine, which blocks oxidative burst response in granulocytes in vivo [27]. When histamine dihydrochloride was injected, the ameliorating effect of phytol on arthritis severity was reversed (Figure 5G), further highlighting the importance of ROS for the therapeutic effect.

### Efficiency of Phytol Is Comparable to Standard Treatments

**Figure 6:**
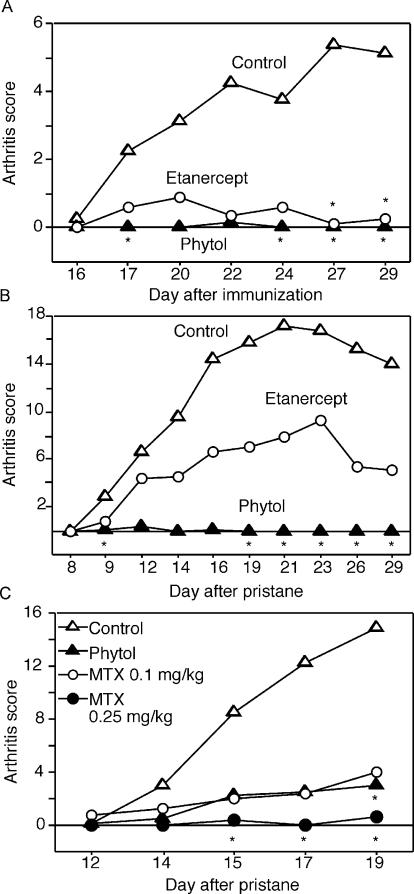
Phytol Is as Effective as or More Effective than Standard Treatments for RA (A, B) Comparison of efficacy of phytol and etanercept in (A) CIA (*n* = 8) and (B) PIA (*n* = 7) in DA.*Ncf1* ^DA^ rats. 200 μl of phytol and 0.4 mg/kg etanercept in a total volume of 200 μl was injected (SC) on days 8, 10, and 12 after disease induction. (C) Efficacy of phytol and MTX. MTX (IP) and phytol (SC) was injected on days 8, 10, and 12 (control *n* = 9, phytol *n* = 9 and MTX *n* = 5) after PIA induction in DA.*Ncf1* ^DA^ rats. *p < 0.05.

MTX is often the first choice of therapy for RA and is in many cases used in combination with blockers of tumour necrosis factor-α, a key cytokine in RA [28–30], such as etanercept. We first compared the efficiency of phytol and etanercept in the CIA model, in which tumour necrosis factor-α blockers previously have shown good effects in rats [31]. Both phytol and etanercept (administered SC on days 8, 10, and 12) were highly effective in reducing CIA (Figure 6A). In the PIA model, the prevention mediated by phytol was even more pronounced than that of etanercept (Figure 6B). Furthermore, no significant reduction of disease severity could be seen after a single etanercept administration at this stage of disease, while phytol significantly lowered the disease scoring (unpublished data). Also in comparison to MTX, phytol was valid as a potential therapeutic agent (Figure 6C).

### Phytol Inhibits Arthritogenic T Cells

Phytol was effective at different stages of arthritis. Thus we wanted to investigate if phytol actually operates in stage-specific ways rather than through long-term effects. It is possible to address this question since PIA is transferable by T cells. We have shown that when spleen cells are collected 10 d after pristane administration and cultured in presence of concanavalin A before transfer to recipient rats, the arthritogenic cells are CD4^+^ αβT cells [22] introducing a good tool for studying the effect of increased oxidative burst on these cells.

**Figure 7:**
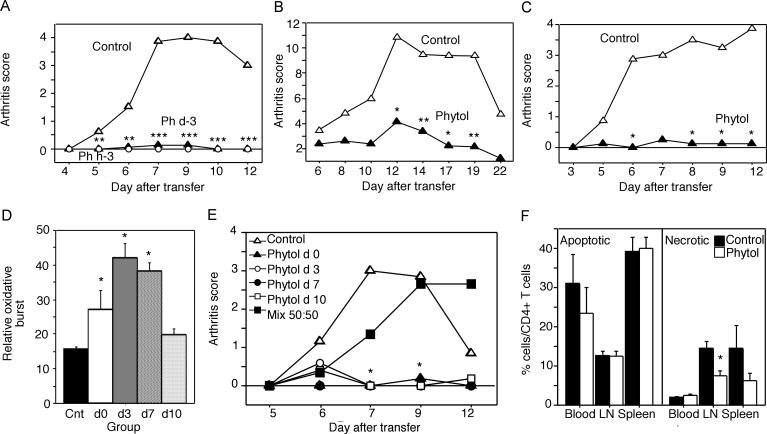
Phytol Inhibits Arthritogenic T Cells In Vivo (A) Phytol (Ph) prevents adoptive transfer of arthritis when injected into the recipient DA.*Ncf1* ^DA^ rat before onset. Phytol was injected (200 μl SC) in the recipient 3 d or 3 h before transfer of arthritogenic T cells from pristane injected DA.*Ncf1* ^DA^ rats (control, *n* = 8; phytol, *n* = 16). (B) Phytol ameliorates arthritis also when injected in recipient DA.*Ncf1* ^DA^ rats after disease onset. Phytol was injected (200 μl SC) in the recipient rats days 5 and 8 after transfer of arthritogenic T cells (control *n* = 7 and phytol *n* = 8). (C) Phytol inhibits arthritogenic T cells when injected in the donor DA.*Ncf1* ^DA^ rat. Spleen cells from phytol treated (500 μl SC day −5) rats were taken day 10 after pristane injection, transferred into naïve DA.*Ncf1* ^DA^ rats and the effect of phytol on the development of arthritogenic T cells were followed (*n* = 8). (D, E) Phytol has a rapid inhibitory effect on arthritogenic T cells. The time-point for the effect of phytol was titrated by administration of phytol (500 μl SC) on different days after pristane injection (day 0). Day 10 after pristane, donor spleens (DA.*Ncf1* ^DA^) were taken and analyzed for oxidative burst of granulocytes (*n* = 3) (D) and the cells transferred into naïve DA.*Ncf1* ^DA^ recipient rats. The rats were then monitored for arthritis symptoms (E). Cells from rats treated day 0 and control rats were also mixed (50:50) to investigate a possible dominating effect of phytol treatment (control [Cnt], *n* = 6; phytol, *n* = 5; and mix, *n* = 3). (F) Phytol does not induce acute cell death. Percentage of apoptotic (Annexin V+ and PI−) and necrotic (Annexin V+ and PI+) CD4^+^ cells ex vivo 3 h after phytol (*n* = 5). *p < 0.05, **p < 0.01, ***p < 0.001.

To address the question of whether phytol has a preventive or therapeutic effect on adoptively transferred PIA, we pretreated the recipient rats before or after transfer of arthritogenic T cells. We found equally good preventive results against arthritis with these treatments (Figure 7A and 7B). Additionally, we wanted to know whether phytol had an effect on in vivo activation of arthritogenic T cells. Therefore, donor rats were treated with phytol prior to spleen isolation. An almost complete blockage of disease could be seen in rats transferred with cells from phytol-pretreated (day −5) rats (Figure 7C), suggesting a phytol-mediated inhibition of arthritogenic T cells.

To determine when this inhibitory effect on the arthritogenic T cells is important, we injected phytol in the donor at different time points after pristane, i.e. at days 0 (following pristane injection), 3, 7, and 10 (3 h before sacrifice). When donor splenocytes were analyzed for oxidative burst on day 10, rats given phytol up to 3 d before sacrifice had a higher level of intrinsic oxidative burst (without PMA stimulation in vitro) (Figure 7D). No difference in oxidative burst was seen after PMA stimulation, possibly due to the proximity to arthritis onset (unpublished data). Interestingly, none of the rats receiving cells from phytol-treated donors developed arthritis, even if the phytol treatment was given as late as 3 h before sacrifice (Figure 7E). Together with the observation that no increase in oxidative burst capacity occurred in spleen cells from these rats, this argues that ROS-producing cells affect the arthritogenic T cells in vitro, or that the NADPH oxidase activation is transferred to the recipient. However, when oxidative burst was measured in recipients 2 d after transfer, no differences were seen (unpublished data) and when the recipient rats were reimmunized with pristane, all groups developed arthritis (unpublished data) excluding transfer of regulatory T cells.

To further exclude the influence on T cells by oxidative burst-producing cells in vitro, we mixed cells, before culture, from control rats and rats treated on day 0. Rats transferred with cells from this mix did develop arthritis (Figure 7E), although only half the number of arthritogenic T cells were transferred, suggesting that this is the dominant phenotype. To exclude an apoptosis-inducing effect, we analyzed blood, spleens, and draining LNs for cell death 3 h after phytol injection. Apoptosis did not increase after phytol, in agreement with data from 5 d after treatment. Phytol even seemed to protect from necrosis (Figure 7F), excluding an acute cell death-inducing effect of the treatment. This experiment clearly shows that phytol exercises a rapid regulatory effect on arthritogenic T cells in both priming and secondary activation stages in vivo*.*

## Discussion

Our results showing that arthritis suppression is mediated by increased oxidative burst in vivo open up a chronic inflammatory disease pathway that is possible to target therapeutically. This new therapeutic approach is based on reversing a phenotype caused by a genetic polymorphism responsible for increased severity of arthritis in both rats and mice [7,32], increasing the possibility that it also operates similarly in humans. The ROS-inducing compound phytol showed comparable or better therapeutic effects in comparison with two of the major drugs used for RA today, i.e., etanercept and MTX, further confirming its possible therapeutic efficacy. Phytol successfully ameliorated arthritis not only in rats with low ROS production but also in rats with normal oxidative burst response, in rats with a different genetic background, and in different models such as PIA, CIA, NOCIA, and adoptive transfer models. Taken together these data suggest that several subtypes of the heterogeneous disease RA could be treated using this approach.

Generally, ROS is thought to play a major role in defence against invading pathogens. A complete lack of ROS due to *Ncf1* deficiency results in chronic granulomatous disease in both mice [33] and humans. Patients with chronic granulomatous disease suffer not only from a granulomatous inflammatory disease but also from increased susceptibility to infections. The observed increased arthritis severity in low burst-responding rats could be influenced by increased infection susceptibility. We believe that this is not the case, as we did not observe any pathogenic infections in the rats. Also preliminary data suggest that mice with an *Ncf1* mutation showed similar arthritis susceptibility in both conventional and SPF conditions (unpublished data). Most likely, the investigated *Ncf1* polymorphism have different effects as compared with the complete *Ncf1* deletion causing chronic granulomatous disease.

Recently ROS has been thought not only to act on invading pathogens but also to play an important regulatory role in the immune system. Although granulocytes are the main ROS-producing cell type, a preventive effect on arthritogenic T cells could clearly be seen after treatment. This preventive effect could be mediated at several different stages. It has been shown that coincubation of T cells with ROS-producing granulocytes inhibits cytokine production [34]. Also, T cell expansion [35] and apoptosis [36] are affected by altered levels of oxygen radicals. The increased ROS production seen after phytol treatment could in addition change the general oxidation status of T cells and thereby mediate suppression. Oxidation of proteins on cell surfaces has been shown to be crucial for function, e.g., the protein LAT (linker for activation of T cells) is highly sensitive to redox alterations, resulting in lower T cell receptor signalling after oxidation [37]. A reducing environment has also been shown to be important for T cell proliferation [38]. T cells have themselves been proposed to express the components of the NADPH oxidase complex and to produce ROS [39]. We measured an increased oxidative burst in T cells after phytol treatment, but to a dramatically lower level than that of granulocytes. This increased ROS could be a result of ROS leaking from surrounding granulocytes or produced by other oxidative burst mechanisms independent of Ncf1. We could not exclude, however, the possibility that the T cell oxidative burst has an effect on autoimmunity by itself. T cells are likely to be regulated by ROS produced extracellularly from other cells, such as the Ncf1-dependent activities of antigen-presenting cells and granulocytes, as well as by internal mitochondrial activities. For example, protein tyrosine phosphatases, regulators of a wide array of cellular signalling pathways, can be inactivated by oxidation [40], which is interesting because a polymorphism in *PTPN22* has been shown to be associated with several chronic inflammatory diseases, including RA [41]. This mutation leads to a more potent negative effect on T cell receptor signalling akin to what can be envisioned by a reduced cysteine in the combining site of the protein tyrosine phosphatases.

ROS can also directly act as a second messenger in signalling pathways—for example, in the Raf-1/extracellular signal-regulated protein kinase signalling cascade [42]—or the effect of phytol could be mediated indirectly. It has been shown that oxidized phospholipids could block Toll-like receptor-mediated activation of dendritic cells [43] and macrophages [44], limiting their capacity to stimulate T cells, thereby suggesting a regulatory feedback mechanism.

The increase in oxidative burst after phytol was not restricted to one anatomical compartment and was observed in blood, BM, spleen, and LNs. Thymus could also be affected by this treatment. Altered oxidation status could affect the depletion of autoreactive T cells and the activation threshold of autoreactive T cells, resulting in a lower level of autoreactivity. Ultimately, phytol affected development of arthritogenic T cells very rapidly. As early as 3 h after phytol injection it was no longer possible to transfer disease from pristane-injected rats. The arthritis-preventive effect in adoptive transfer probably operates through down-regulation of arthritogenic T cells and is not mediated by ROS-producing cells in vitro or in the recipient, since the mix of treated and untreated cells transferred arthritis, and no alterations of oxidative burst occurred in the recipient after transfer. The early preventive effect seen after phytol is likely not mediated via induction of oxidative burst in T cells, since the effect is lost in phytol-primed T cells cultured together with arthritogenic T cells. These findings also exclude transfer of regulatory T cells, since recipients developed PIA in a normal manner.

These data provide a new strategy to prevent and ameliorate established arthritis through a previously little-studied pathway involving a protective oxidative burst. The treatment was effective in different arthritis rat models, different rat strains, and at different stages of the disease regulated by different genes and mechanisms. Thus, phytol represents a promising new class of pharmaceuticals to further investigate for treatment of chronic inflammatory diseases.

## Supporting Information

Figure S1 Anti-CD3 Stimulation of T Cells from Naïve and Phytol-Injected DA.Ncf1DA Rats Cells were taken from different anatomical sites 5 d after phytol injection. Following lysis of red blood cells, remaining cells were stained for T cell receptor with R73 then incubated with plate-bound anti-CD3 (G4.18) in the presence of DHR-123. Cells were analysed for R-123 by FACS (*n* = 5). *p < 0.05.
(194 KB JPG)

## Abbreviations

BM: bone marrow
CIA: collagen-induced arthritis
CII: collagen type II
COMP: cartilage oligomeric matrix protein
IP: intraperitoneal(ly)
LN: lymph node
MTX: methotrexate
NOCIA: nonoil collagen-induced arthritis
PIA: pristane-induced arthritis
PMA: phorbol 12-myristate 13-acetate
RA: rheumatoid arthritis
ROS: reactive oxygen species
SC: subcutaneous(ly)
